# Distribution and fixed-precision sampling plans for diamondback moth (Lepidoptera: Plutellidae), on winter–spring cabbage

**DOI:** 10.1093/jee/toad156

**Published:** 2023-08-20

**Authors:** Derick Nomuh Forbanka, Mxolisi Arnold Stemele, Lelethu Unathi-Nkosi Peter Heshula, William Diymba Dzemo, Daniel Ashie Kotey, Amon Taruvinga, Pfarelo Grace Tshivhandekano

**Affiliations:** Department of Zoology and Entomology, University of Fort Hare, Alice 5700, South Africa; Department of Zoology and Entomology, University of Fort Hare, Alice 5700, South Africa; Department of Zoology and Entomology, University of Fort Hare, Alice 5700, South Africa; Department of Biological and Environmental Sciences, Walter Sisulu University, Mthatha 5117, South Africa; CSIR–Plant Genetic Resources Research Institute, P.O. Box 7, Bunso Eastern Region, Ghana; Department of Agricultural Economics and Extension, University of Fort Hare, Alice, South Africa; Department of Zoology and Entomology, University of Fort Hare, Alice 5700, South Africa

**Keywords:** insecticide application, sampling plan, economic injury level, action threshold

## Abstract

The diamondback moth (*Plutella xylostella* L.) is the most destructive insect pest on cabbage (*Brassica oleracea* var. *capitata* L.). Infestation by this pest usually results in the indiscriminate use of insecticides by farmers due to a lack of sampling plans for this pest. Sampling plans for *P. xylostella* management decisions on winter–spring cabbage in the Eastern Cape Province of South Africa were developed, through population monitoring that comprised weekly counts of immature stages of *P. xylostella* on 60 plants for 11 wk each during the winter and spring seasons. The mean density–variance relationship was used to describe the distribution of the pest, and number of infested plants was used to develop a fixed-precision sampling plan. All plant growth stages preceding maturation were vulnerable to *P. xylostella* damage resulting in yield losses. A high aggregation of *P. xylostella* on cabbage was observed in spring than in winter. The average sample number to estimate *P. xylostella* density within a 15% standard error of the mean was 35 plants. Furthermore, the estimated plant proportion action threshold (AT) was 51% with density action thresholds of 0.50 and 0.80 for spring and winter, respectively. Fitting *P. xylostella* cumulative counts in the winter and spring sampling plans resulted in 100% and 45% reduction in insecticide treatments. The similarity of sample size and ATs between both seasons provides evidence that a single sampling plan is practical for all cabbage growing seasons. The similarity of the estimated ATs to those acceptable in established integrated pest management programs indicates reliability.

## Introduction

Cabbage (*Brassica oleracea* var. *capitata*) is a dense leaved vegetable crop consumed raw, cooked, or processed. In South Africa, cultivation of cabbage on a commercial basis is done near markets in periurban areas, while small-scale farmers grow cabbage for subsistence in rural areas (Charleston 1998). Small-scale farmers often experience severe yield losses in their fields due to insect pests, diseases, and abiotic factors related to prevailing climate and agronomic practices. In the Eastern Cape Province of South Africa, cabbage diseases and many insect pest species are prevalent during summer, the prime rainfall season (Stemele 2016). Hence, subsistence cabbage is mostly cultivated during the winter and spring seasons, that is, winter–spring cabbage.

Globally, the diamondback moth (DBM), *Plutella xylostella* L. (Lepidoptera: Plutellidae), is the most destructive insect pest of *Brassica* crops (Talekar and Shelton 1993, Furlong et al. 2013). The estimated annual global cost of *P. xylostella* management, crop damage, and yield losses is estimated at US$4–5 billion (Zalucki et al. 2012). *Plutella xylostella* populations persist all year round in South Africa and cause 16–46% yield loss in insecticide-free cabbage in small-scale production systems (Stemele 2016). However, higher yield losses between 40% and 51% have been reported elsewhere (Grossrieder et al. 2005, Ayalew 2006). In response to the risks of severe crop losses, many farmers rely on calendar and prophylactic sprays using a wide range of chemical insecticides. Many such insecticides are broad-spectrum formulations of carbamates, organochlorines, and triazine (Quinn et al. 2011).

Insecticides are important damage-reducing inputs in the agroecosystem (Lansink and Silva 2004). However, many farmers do not comply with recommended application rates and frequencies for commonly used insecticides (Williamson et al. 2008). In most instances, this lack of compliance results in detrimental effects on the environment (Furlong et al. 2004), selection for insecticide-resistant strains of insect pests (Sereda et al. 1997), and the elimination of natural enemies (Kfir 2004). Moreover, the indiscriminate use of insecticides raises concerns about the health of farmworkers and the safety of consumers. Many farmers only appreciate and are familiar with the acute action of chemical insecticides on insect pests. Indeed, Singh et al. (2018) emphasize that insecticides must only be used based on need when crop damage is unavoidable.

During the last decade, cabbage production has seen a steady annual increase of 4.04 metric tons (mt) with a high surplus for export (Statistic South Africa 2021). Export markets however have stringent regulations regarding maximum insecticide residue limits on produce according to the Food and Agriculture Organization Codex Alimentatius online database. These stringent maximum residue levels for cabbage production and exportation therefore call for research to support the production efforts of farmers by providing monitoring tools to regulate the use of insecticides and minimize the danger of insecticide residues. One way to do this is to provide effective alternatives to insecticide application and develop integrated pest management (IPM) programs. A fundamental step in developing and implementing an IPM program is to incorporate alternate pest control strategies followed by a stepwise introduction of chemical insecticides.

IPM programs sustain high crop yields (Furlong et al. 2008) by reducing and maintaining insect pest populations below economic injury levels (EILs) without detrimental effects on the environment (Francis et al. 2005). In many developed countries, IPM programs have outlined procedures and control strategies for primary crop pests. In such IPM programs, farmers make use of action and economic thresholds that depend on a protocol-based assessment of the pest population in the field (Binns et al. 2000, Southwood and Henderson 2016). This informs the decision-making process concerning the necessity to implement an insect pest control strategy (Furlong et al. 2004, Hamilton and Hepworth 2004). Pest population assessments usually require pest monitoring using established sampling plans (Binns et al. 2000, Southwood and Henderson 2016).

Sampling plans are multipurpose decision-making tools used in IPM for the accurate estimation of pest populations in the field (Panthi et al. 2021) to avoid unnecessary insecticide application. The use of these plans typically requires fewer samples and lesser time to estimate pest populations compared to whole-field scouting methods of sampling (Southwood and Henderson 2016). In addition, such tools are used to evaluate the effectiveness of a pest control strategy (Pezzini et al. 2019). The determination of the distribution of an insect pest is a prerequisite for the implementation of a sampling plan (Binns et al. 2000). Field insect pest counts are usually modeled to determine the distribution parameters and aggregation indices specific to a pest in a given crop (Binns et al. 2000). The distribution parameters obtained can then be modeled with mathematical equations to determine the number of sampling units required to estimate a pest’s mean field density (Binns et al. 2000). Fixed-precision sampling plans usually define the sample unit, sample size, threshold densities, and timing for executing an insect control strategy in the field. This is usually based on pre-established precision levels that inform the decision-making process in pest management (Hodgson et al. 2004). Thus, there is a considerable opportunity to improve the monitoring and management decision of *P. xylostella* on cabbage. However, in South Africa, there are no sampling plans or threshold levels defined for *P*. *xylostella* on cabbage despite the economic importance of this insect pest. Hence, the objectives of this study were to determine the temporal distribution of *P. xylostella* on cabbage and develop a fixed-precision sampling plan for the Eastern Cape Province of South Africa.

## Materials and Methods

### Study Site and Cabbage Production

The field trials were conducted on a communal farm in Keiskamahoek (−32.757N, 27.079E; 530 m above sea level), in the Eastern Cape Province, South Africa. Like all communal farms in South Africa, this farm is owned by the government but managed by local (tribal) authorities (Brunce 2018). The study site is located within the Drought Corridor Ecological Zone that is characterized by cold winter (−2 to 22 °C) and moderate spring (15 to 25 °C). Cabbage cultivation takes place during winter (June–August) and spring (September–November) seasons. Despite the availability of heat-tolerant cabbage cultivars, small-scale farmers of the Eastern Cape Province of South Africa usually concentrate their efforts on maize cultivation during the summer and autumn seasons (December–April, sometimes extending to May). The cabbage varieties, STAR3301 and Green Coronet (Starke Ayres, South Africa), were selected to be used in the field trials. The STAR3301 has been adapted for the cold winter season, while Green Coronet is adapted for the warm spring season.

### Field Layout

A week before transplanting, nitrogen, phosphorus, and potassium were broadcast by hand and incorporated into the soil at a rate of 50, 30, and 40 kg/ha, respectively, using NPK 3:2:1 (25), limestone ammonium nitrate (28%), and potassium chloride (50% K). Seedlings were transplanted to 20 × 10 m plots replicated 3 times for each of the seasons. Each replicate was separated by a 1 m space. Within each plot, a total of 286 seedlings of each of the 2 varieties were planted at a 90 × 80 cm intra- and inter-row spacing. Biobit HP WP: (*Bacillus thuringiensis* var. *kurstaki* [*Btk*] 32,000 IU/mg, Valent BioSciences, South Africa) was applied on the cabbage seedlings to maintain low insect pest infestation. This bioinsecticide was applied weekly after irrigation at the manufacturer’s recommended dose of 250 g/ha using a manual flat-fan nozzle GS0341 knapsack sprayer (Green Industrial Supplies, South Africa). Irrigation of the trial plots was conducted biweekly using overhead sprinklers. Nitrogen (NPK 3:2:1 (25)) top dressing of 150 kg/ha was applied at 4 and 8 wk after transplanting.

### Sampling

Data were collected during the 2014, 2016, and 2019 winter–spring seasons. Due to logistics, no data were collected in 2015, 2017, and 2018. The data collected in 2014 and 2016 were used to develop the sampling plans, whereas data collected in 2019 were used to evaluate the sampling plans. The experiments started a week after transplanting until 1 wk before harvest to include all crop stages as classified by Andaloro et al. (1983). Once a week, 60 (20 × 3) plants from each of the cabbage varieties were selected for sampling. The sample size of 60 plants was chosen to estimate insect population density with high precision (Hamilton et al. 2009). Sampling was conducted for 11 wk for each of the seasons starting from June for winter and September for spring.

The sampling of individual plants involved the use of a sampling sheet drawn on paper. The sampling sheet comprised 22 × 13 grids with each grid representing an individual plant (sampling unit) in the plot. Before sampling, 20 grids were selected randomly and marked on the sampling sheet. This technique reduced the chances of selecting the same plant within 2 wk (i.e., an approximate development period for *P. xylostella*) (Sow et al. 2013, Stemele 2016, 2017, Machekano et al. 2017). The upper and lower surfaces of outer as well as inner leaves (growing cup) of cabbage plants in the field that corresponded with the selected blocks marked on the sampling sheet were inspected for *P*. *xylostella* infestation. The number of *P. xylostella* on each plant and zero counts in each of the respective grids were recorded. Each sampling sheet represented records of weekly infested and uninfested plants per plot and the *P. xylostella* density per plant, which was later converted to mean weekly insect densities.

### Determination of the Distribution of *P. xylostella* on Cabbage Plants

The mean weekly densities of *P. xylostella* were transformed into Lloyd’s indices of mean crowding (Lloyd 1967) (X*) as shown in equation 1. This index describes the aggregation of insects per plant within an area. It is thus ideal for the assessment of the distribution of insects in the field. Unlike the mean density, the number of sample units (plants) with zero insect counts does not affect the aggregation described by the index (Stabeno et al. 1996). The Lloyd’s index of mean crowding was calculated as follows:


$$X^{*}=\bar{x}+\left(\frac{S^{2}}{\bar{x}}-1\right)$$ (1)

where $\bar{x}$ is the weekly mean density, and *S*^2^ is the sample variance. A high X* indicates high aggregation of *P*. *xylostella* per plant.

Based on IPR (Iwao 1968), the Kuno’s fixed-precision model (Kuno 1969) was used to develop a sampling plan for *P*. *xylostella*. The IPR describes the distribution of *P. xylostella* as a relationship between the index of mean crowding (X*) and the mean density $\bar{x}$ calculated as:


$$X^{*}=\;\alpha +\beta \bar{x}$$ (2)

where *α* is an intercept and *β* the slope. The intercept is an index of contagion that describes the grouping or dissociation of individual insects on plants. When *α* < 0, it demonstrates dissociation, when *α =* 0, it indicates a single individual, and when *α* > 0, it signifies grouping of individuals per plant. The slope (*β*) characterized the distribution with *β* < 1, demonstrating a uniform pattern; *β* = 1, indicating a random pattern; and *β* > 1, representing an aggregated spatial pattern.

### Construction of the Sampling Plans for P. xylostella on Cabbage Plants

The minimum sample size (*n*_*min*_) required to predict *P*. *xylostella* density within 0.15 precision (*D =* 15%) was calculated as follows:


$$n_{min}=\;\frac{\left(\alpha +1\right)}{\bar{x}+(\beta -1)}÷D^{2}$$ (3)

where *α* and *β* are the IPR parameters (equation 2), and *D* is the level of precision expressed as a standard error of the density in the field divided by the mean density, and is calculated as:


$$D=\frac{S}{\sqrt{n}}÷\bar{x}$$ (4)

where *s* represents the standard deviation and *n* denotes the number of samples. The stop lines of the sampling plan were calculated as:


$$T_{n}=\frac{\left(\alpha +1\right)}{D^{2\;}-(\beta -1)/n_{min}}$$ (5)

where *T*_*n*_ is the cumulative number of *P. xylostella* in a sample size of *n*_*min*_ sampling units (plants). The sampling chart obtained from a regression of the *n*_*min*_ against *T*_*n*_ estimates the optimum sample size required to predict pest density at the predefined precision level.

### Validation and Evaluation of the Sampling Plans

The sampling plan adoption and application for decision-making in pest management depends on the precision of the plan (Galvan et al. 2007). The operating characteristic (OC) curve and the average sample number (ASN) were used to assess the accuracy and cost-effectiveness of the plan. The OC curve is the probability that the sampling plan recommends an appropriate decision not to control when the proportion of infested plants remain below an action threshold (AT). To obtain an OC curve, we calculated the binomial probability distribution of the average number of infested plants per week in Microsoft Excel. The resulting probabilities were fitted in the Gompertz function:


$$\text{OC}=ae^{be^{xc}}$$ (6)

where *a* is an asymptotic fixed constant, *x* is the population density, *c* is the point of inflection, *e* is the Euler’s number (2.718), and *b* represents the steepness of the slope.

Calculated ASN values provide an optimum sample size expected to predict the pest density with a specified level of precision. To obtain ASN, the number of infested plants was transformed into a proportion (*p*) of infested plants using a regression equation with the mean density $\left(\bar{x}\right)$. Subsequently, the density action threshold (*T*_*m*_) was converted into a proportion of infested plants (*T*_*a*_) by fitting the slope (*β*) and intercept (*α*) in Nachman’s model expressed as:


$$p=1-e^{\left(-\alpha {\bar{x}}^{\beta }\right)}$$ (7)

The ASN was obtained by fitting the observed and derived proportion of infested plants in the Gaussian function (equation 7) expressed as:


$$\text{ASN}=ae^{-\left(-\frac{{\left(x-b\right)}^{2}}{2c}\right)}$$ (8)

where *a*, *b*, and *c* are >0; *a* is the height of the curve’s peak, *b* represents the position of the center of the peak, and *c* is the standard deviation. The density action threshold was also obtained by fitting the mean density with the observed plant proportions in the same equation.

As an extension of the validation of the sampling plans, the cumulative weekly number of insects was plotted into the sampling plan. The decision to treat or not to treat depends on the cumulative mean density position relative to the stop lines. Treatment is required if the plot point crosses the stop line. The statistics software package, PAST ver. 3.22 (Hammer et al. 2001) was used to perform all the analyses.

## Results

### Distribution of P. xylostella During Spring and Winter Seasons

**Fig. 1.**
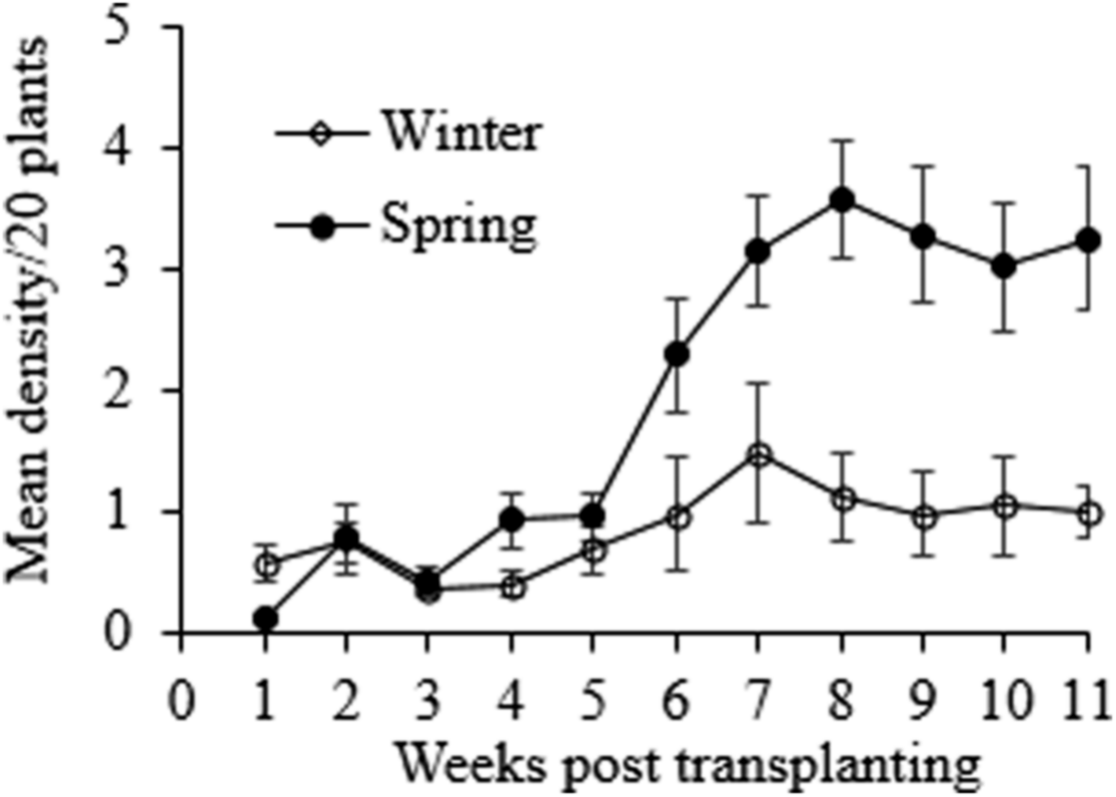
Weekly mean density of *Plutella xylostella* on winter (○) and spring (●) cabbage.

**Fig. 2.**
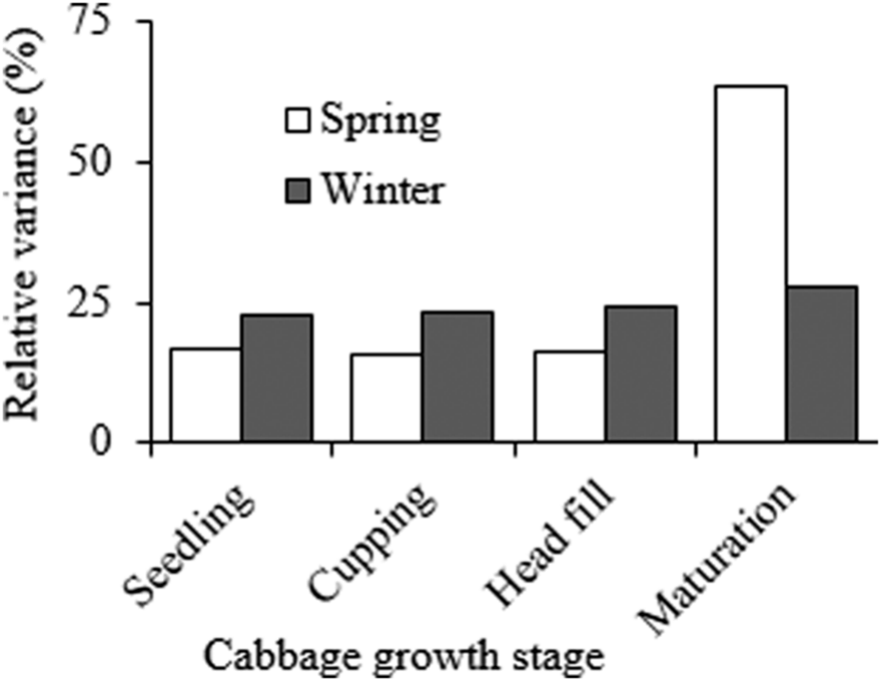
Relative variance of *Plutella xylostella* infestation between different plant growth stages in winter (shaded) and spring (unshaded) cabbage.

The infestation during winter remained low throughout the season. However, during spring, *P. xylostella* infestation was initially low, early in the season. It however increased and attained a peak 8 wk post-transplanting (Fig. 1). This dynamic infestation pattern matched the plant growth stages, with a low mean density occurring during seedling and cupping, from 1 to 5 wk post-transplanting (Fig. 2). The mean density rapidly increased from week 6 and remained high during head filling and early maturation. The mean density for winter and spring samples was 0.47 (range 0.3–0.8) and 1.98 (range 0.1–3.5), respectively. The relative variance remained below 25% during the seedling, cup formation, and head filling stages, which showed that these are preferred *P. xylostella* feeding stages, thus ideal for *P. xylostella* sampling (Fig. 2).

**Fig. 3.**
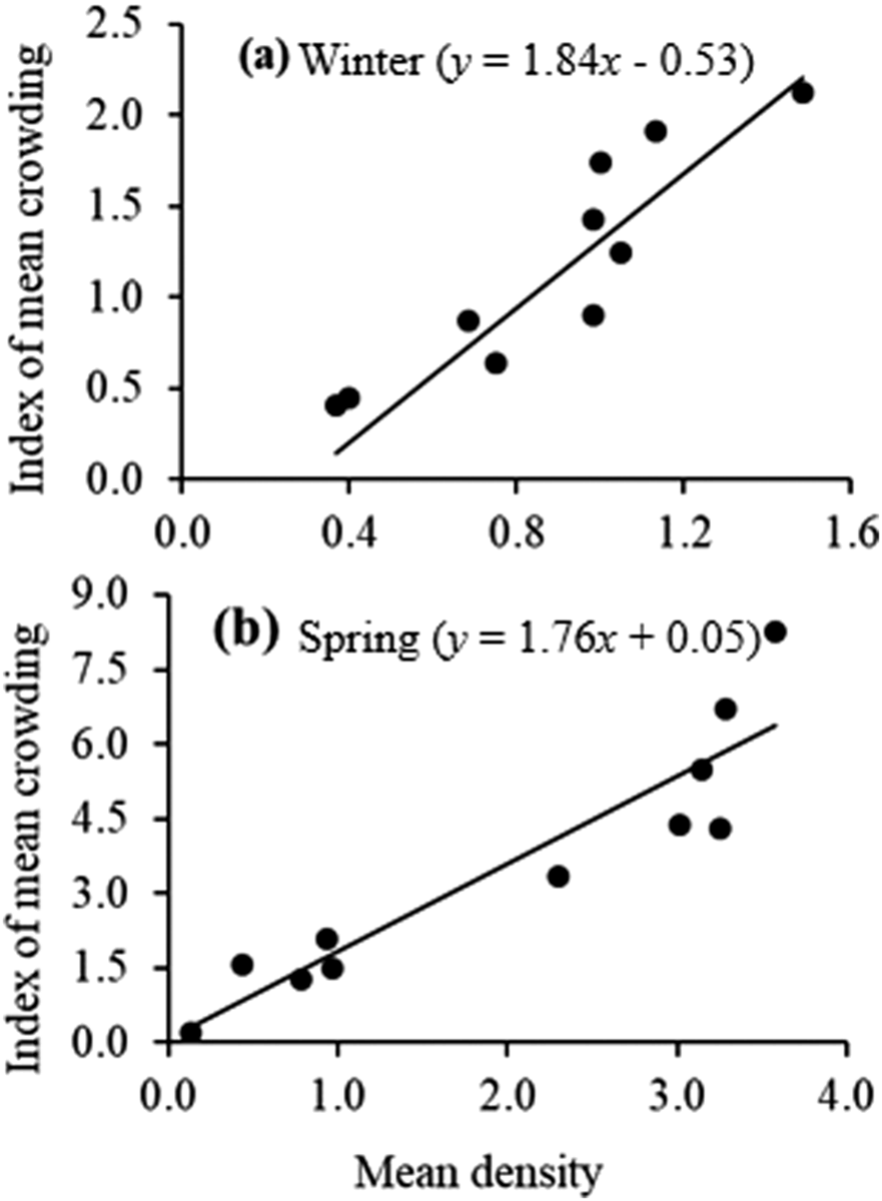
Iwao’s patchiness regression plots of *Plutella xylostella* during (a) winter (*y* = 1.84*x* − 0.53) and (b) spring (*y* = 1.76*x* + 0.05).

Based on the Iwao’s patchiness regression (IPR) model, the mean density indices of crowding were significantly related during winter (X* = 1.59*x* − 0.22; *r*^2^ = 0.82) and spring (X* = 1.38 *x* + 0.29; *r*^2^ = 0.95) (Fig. 3). The slope values were greater than unity indicating an aggregated distribution. The negative intercept value of the winter samples suggested that dissociation prevents grouping, as explained by the low mean crowding during winter (1.14 ± 0.11, range 1.14–2.12) compared to spring (3.0.4 ± 1.08, 1.07–5.87).

### Fixed-Precision Sampling Plans for P. xylostella on Cabbage

**Fig. 4.**
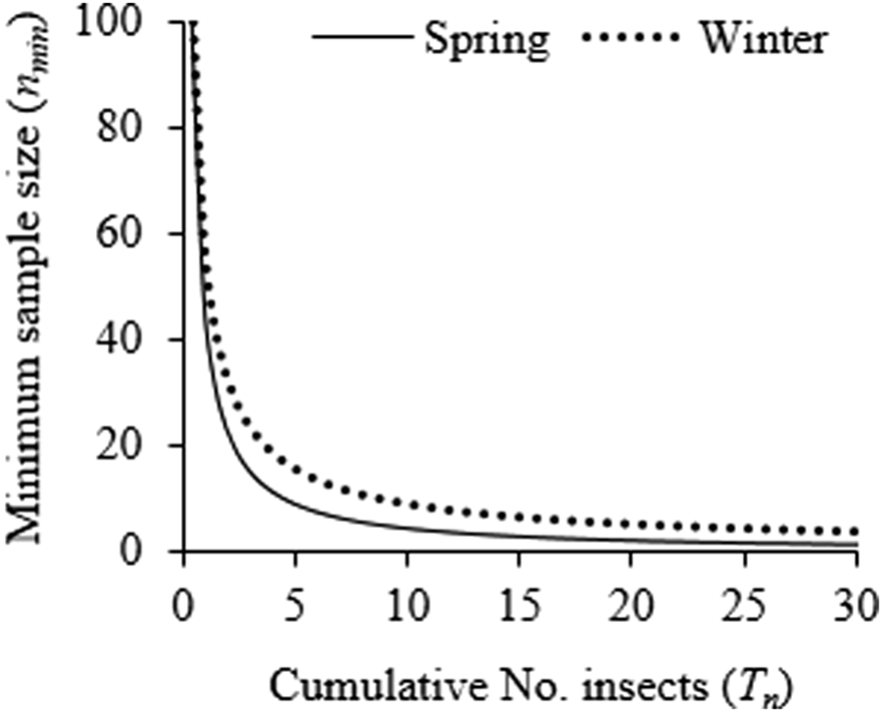
Sampling plans for *Plutella xylostella* at 0.15 and 0.20 levels of precision (*D*) during winter (dotted line; *n* = 58*x*^-0.820^) and (b) spring (solid line; *n* = 47*x*^−1.013^).

Based on sampling charts, the sample size required to estimate population density increased with decreasing density (Fig. 4). The stop lines of the sampling plan required that sampling be terminated whenever the sample sizes reached 94–484 plants for very low (0.1–0.5) *P. xylostella* densities with a 0.15 level of precision. The sampling chart’s stop lines also indicated that optimal sample sizes of 58 and 47 plants are needed to estimate a single insect per plant within a 15% standard error of the mean during the winter and spring seasons, respectively. Therefore, to calculate the required sample size (*n*) at 0.15 precision, the desired mean (*x*) is to be substituted in the equation *n* = 58*x*^−0.820^ for sampling during winter or *n* = 47*x*^−1.013^ for sampling during spring.

### Accuracy and Cost-Effectiveness of Sampling Plan for P. xylostella on Cabbage

**Fig. 5.**
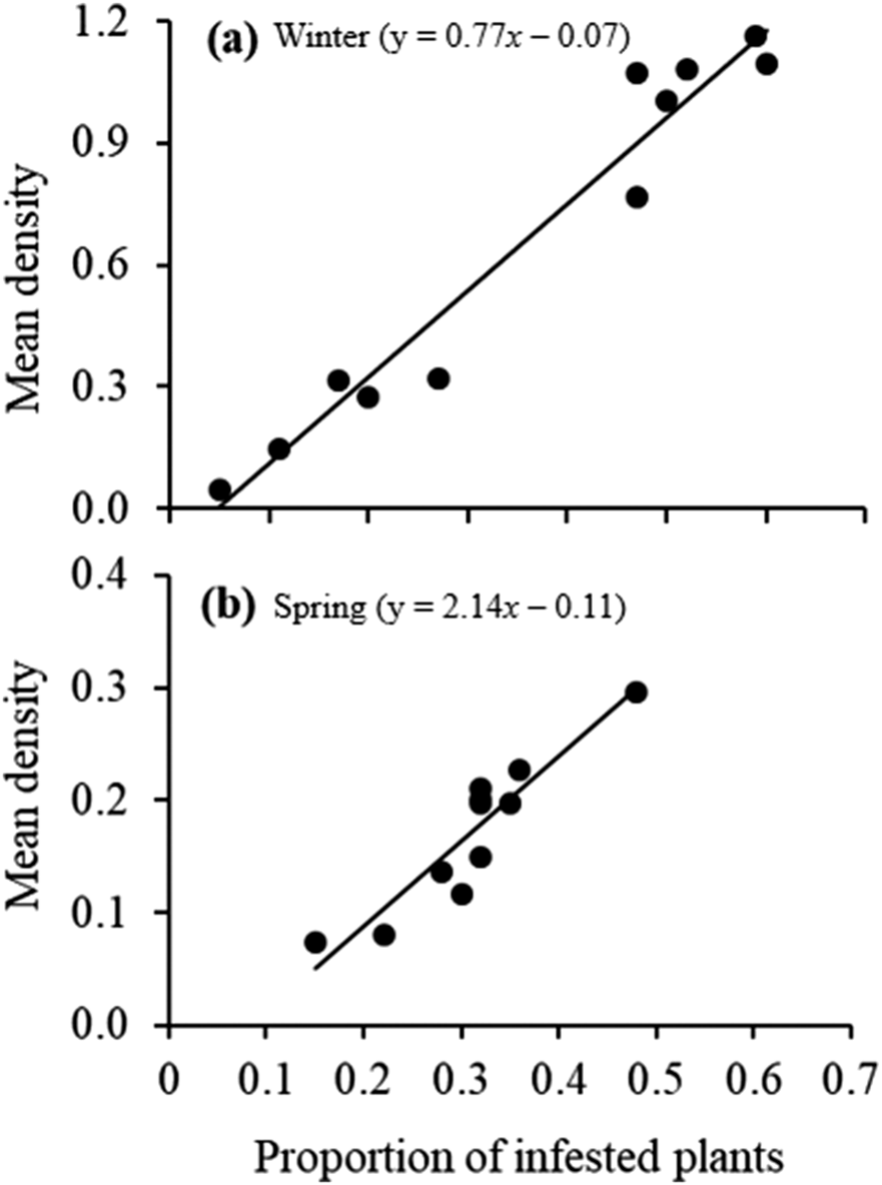
Relationship between mean larval density per plant (*T*_*m*_) and proportion of infested plants (*T*_*a*_) during (a) winter (*y* = 0.77*x* − 0.07; *r*^2^ = 0.87; *P* = 0.00) and (b) spring (*y* = 2.14*x* − 0.11; *r*^2^ = 0.95; *P* = 0.00).

**Fig. 6.**
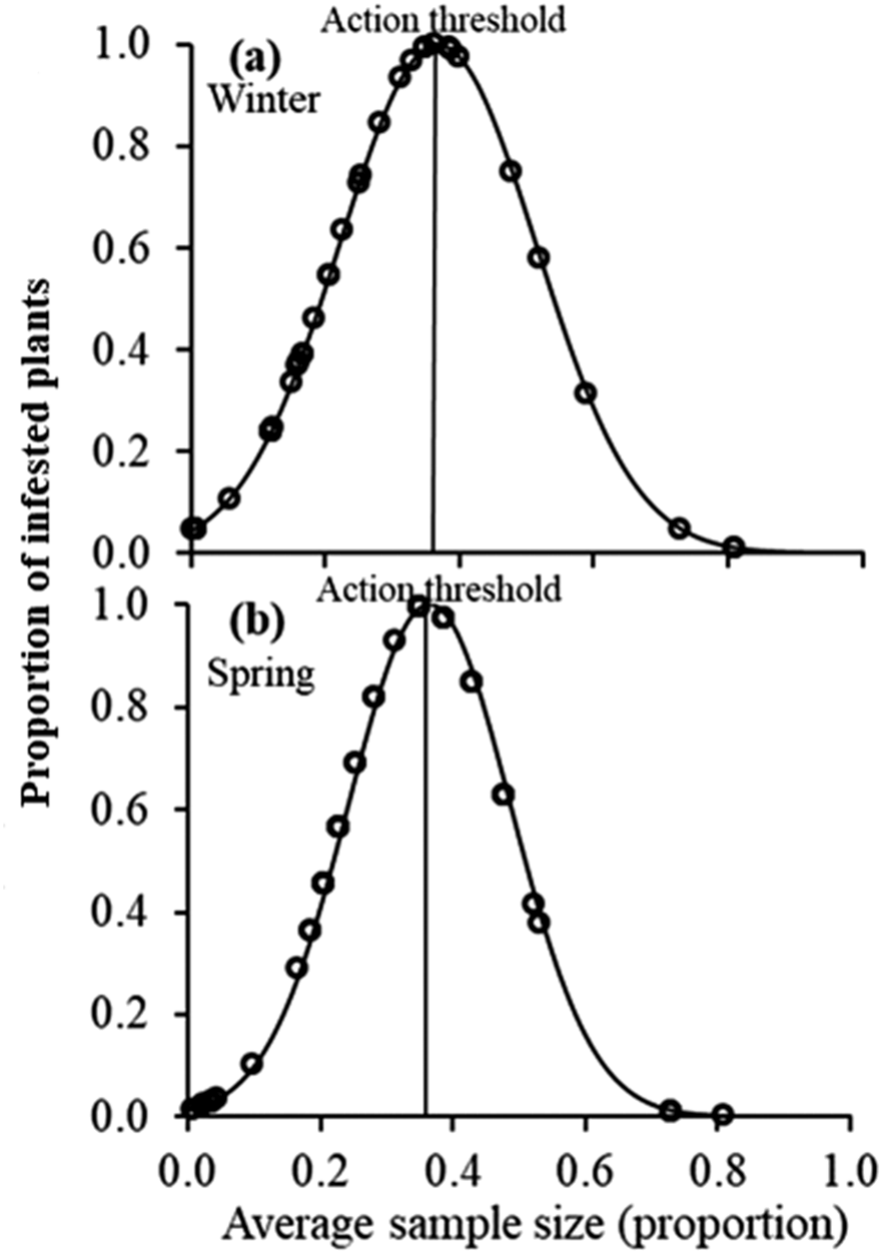
ASN curves for *Plutella xylostella* sampling plans during (a) winter and (b) spring. The vertical lines represent the action threshold (AT_p_) at 0.20 level of precision.

**Fig. 7.**
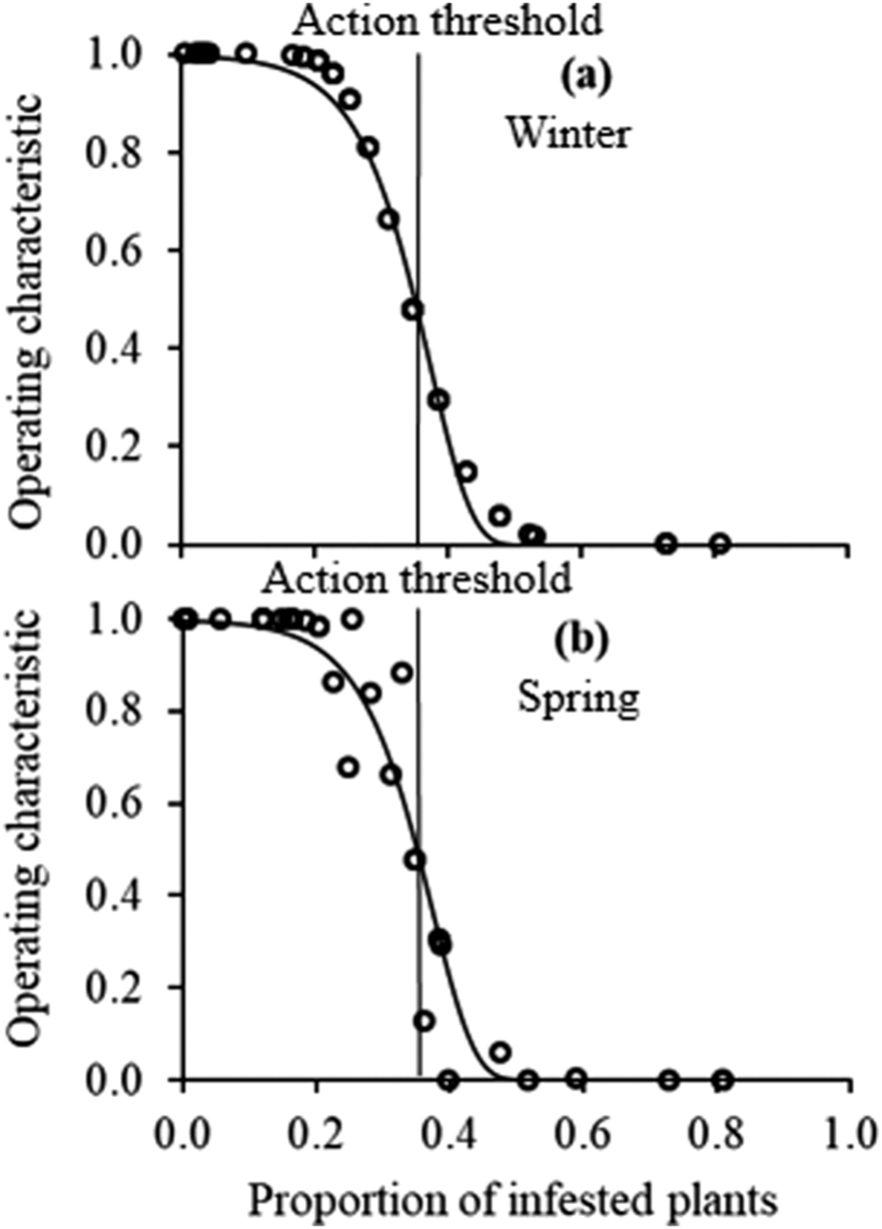
Operating characteristic curves for *Plutella xylostella* sampling plans during (a) winter and (b) spring.

**Fig. 8 .**
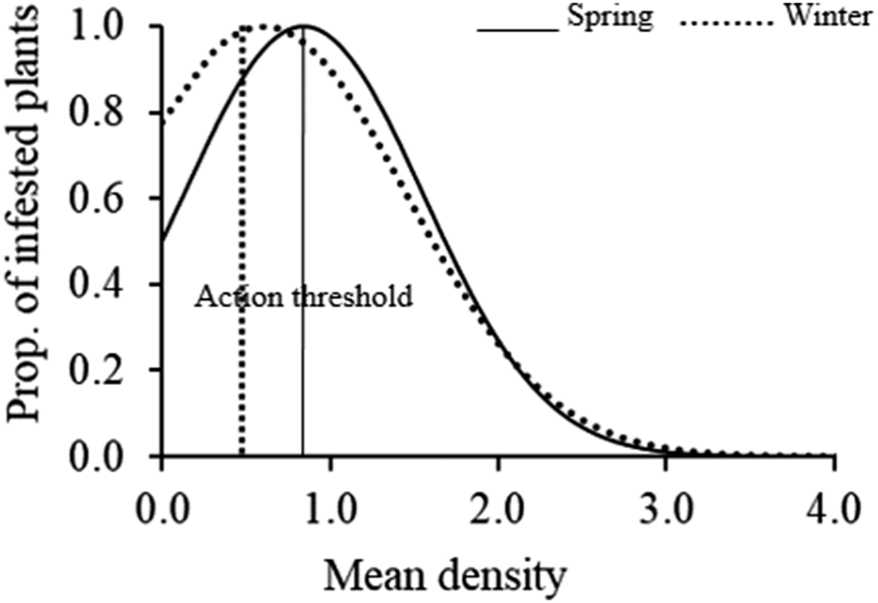
*Plutella xylostella* density action thresholds (ATs, vertical lines) for monitoring *Plutella xylostella* during winter (dotted line) and spring (solid line).

The transformation of the density (*T*_*m*_) into the proportion of infested plants (*T*_*a*_) action threshold using the Nachman model accounted for 87% of the variance during winter (*y* = 0.77*x* − 0.07; *r*^2^ = 0.87; *P* = 0.00) and 95% of the variance during spring (*y* = 2.14*x* − 0.11; *r*^2^ = 0.95; *P* = 0.00) (Fig. 5). The ASN function specified a minimum sample size of 35 plants to sample during winter and spring before deciding the action plan (Fig. 6). The steep slope of the OC curves indicated a high precision of the sampling plans (Fig. 7). The sampling plans were conservative, indicating that they are likely to demand control below the estimated proportion threshold. The average estimated sample size was slightly more than the actual size that corresponds to the estimated 51% infested plant proportion threshold. The insect density action threshold was estimated at 0.50 insects per plant during winter and 0.80 insects per plant during spring (Fig. 8).

**Fig. 9.**
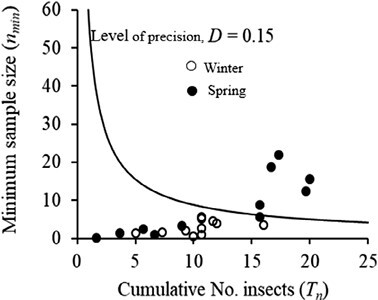
Fixed-precision sampling plans for *Plutella xylostella* fitted with the cumulative number of insects during winter (open circles) and spring (shaded circles).

Fitting the cumulative weekly means of *P. xylostella* articulated no economic efficiency in the current weekly *B. thuringiensis* var. *kurstaki* (*Btk*) application (Fig. 9). The winter sampling plan outperformed the spring plan, demonstrating that no treatment was required during winter and only 6 unavoidable sprays were needed during spring. The winter plan, therefore, reduced the need for insecticide treatments by 100%, whereas the spring plan recommended 45% fewer sprays.

## Discussion

The population of *P. xylostella* on cabbage in the Eastern Cape Province of South Africa has an aggregated distribution as shown by this study. Also, this insect pest does not show any discernible changes in temporal distribution with densities remaining low throughout the winter season, while in spring, there is a gradual increase toward the end of the season. The relative variance in this study showed that plant growth stages preceding head maturation are particularly vulnerable to *P. xylostella* infestation, thus requiring consistent monitoring. This corroborates the findings of Li et al. (2021) who reported that plant growth stages are an influential factor affecting the temporal distribution of *P. xylostella*.

The economic importance of *P. xylostella* stems from its exceptional pest status as a result of genetic diversity, high year-round abundance, high reproductive potential, high genetic elasticity, cosmopolitan distribution, and continuous suppression of its natural enemies by synthetic chemical insects (Furlong et al. 2013, Department of Agriculture, Forestry and Fisheries 2014, Machekano et al. 2017). We therefore used Biobit HP WP: (*Bacillus thuringiensis* var. *kurstaki* [*Btk*] 32,000 IU/mg, Valent BioSciences, South Africa) to suppress *P. xylostella* populations (Dakshina 2013, Stemele 2017). This *Btk* are softest on natural enemies, do not leave residue on the vegetable or environment, and are thus environmentally friendly and compatible with IPM program (Walsh 2005).

Understanding the distribution of an insect pest in field situations is essential for the design of an IPM program (Brenner et al. 1998, Liebhold and Gurevitch 2002) and sampling plan developments (Taylor 1984, Binns et al. 2000, Southwood and Henderson 2016). In addition, knowledge of pest distribution of an insect population is essential in developing sampling plans in particular because factors responsible for variation in pest distribution differ within the field and at landscape and regional scales (Ayalew et al. 2008, Panthi et al. 2021). During winter, *P. xylostella* exhibited no discernible changes in temporal distribution with low density throughout the season. This could be a result of adverse climatic conditions affecting insects during this season. This observation is similar to the results obtained by Mosiane et al. (2003) on canola. On the other hand, with favorable climatic conditions characteristic of the spring season, the population of *P. xylostella* displayed a distinct pattern with a gradual increase in density toward the end of the season as observed by Hasanshahi et al. (2017) in summer–autumn cauliflower.

IPR slope values greater than unity demonstrated an aggregated spatial distribution similar to that reported by previous studies (Chua and Lim 1979, Hamilton and Hepworth 2004, Ayalew et al. 2008). The positive intercept and high mean crowding demonstrating high aggregation during spring could probably be attributed to the egg-laying tendency of adult females. Adult females usually lay eggs in aggregates of 2–8 eggs per leaf (Capinera 2001), with a preference for plants infested by conspecifics (Uematsu and Sakanoshita 1993, Shiojiri et al. 2002). Leaf nutrient level is also an important factor in the distribution of *P. xylostella* in the field (Sarfraz et al. 2009).

The low mean crowding index observed indicated less aggregation during winter, and the negative IPR intercept specified dissociation that prevented grouping. Southwood (1978) suggested that the distribution of insects becomes aggregated at high densities. Therefore, the low aggregation during winter could be attributed to low abundance. Indeed, *P. xylostella* populations exhibit seasonal variation influenced by temperature (Zalucki and Furlong 2008, Sow et al. 2013, Labou et al. 2017). Temperature is known to affect insect oviposition, development, survival, and the number of generations (Marchioro and Foerster 2011, Ngowi et al. 2017).

The sampling charts described by the cumulative number of *P. xylostella* and the minimum sample size observed in this study supported the negative binomial distribution as reported by Chua and Lim (1979). Given the negative binomial distribution of *P. xylostella* populations, Kuno’s sequential sampling model is an appropriate choice for the sampling plan (Hutchison et al. 1988). The sample size increased with decreasing density, and the sampling plan specified large sample sizes to estimate very low *P. xylostella* densities. Nonetheless, these sample sizes were generally low compared to previous studies (Harcourt 1960, Chen and Su 1986, Ayalew et al. 2006, Panthi et al. 2021). This variation could be explained by the use of different methods, empirical equations, location, and the variation in *P. xylostella* strains between geographic regions.

Low *P. xylostella* density during winter required a higher sample size than the spring sampling plan according to sampling charts. The ASN function estimated that a sample size of 35 plants for both seasons was sufficient to estimate *P. xylostella* density within 15% standard error of the mean. Evaluating *P. xylostella* sampling plans in Australia, Hamilton et al. (2006) disqualified 35 plants as enumerative to a presence–absence sampling plan based on low precision. However, in North Korea, Hamilton et al. (2009) demonstrated that sample sizes of 20 plants were acceptable for enumerative sampling plans. Waters et al. (2009) determined the application of a sample size of at least 23 plants within 25% standard error of the mean. These contradictory findings demonstrate that sampling plans defined for an insect pest in one location are not applicable elsewhere (Binns et al. 2000, Panthi et al. 2021). Our results reiterate the postulation that locally defined sampling plans suit the management of local populations. Mo and Baker (2004) endorsed a maximum of 50 plants, which provide more than 95% precision and avoid excessive sampling. The steep OC curve slopes of this study demonstrated the high precision of the sampling plans.

The sampling plan system comprises sample size and thresholds that guide the assessment of pest populations in IPM programs, resulting in reduced insecticide sprays (Picanço et al. 2007). Two types of thresholds apply in pest monitoring: the EIL and AT. The EIL defines the lowest pest density that causes crop injury and economic damage (Stern et al. 1959). The commonly adopted economic threshold of 0.3 *P. xylostella* larvae/plant/week reportedly improves high cabbage yields (Kirby and Slosser 1984, Cartwright et al. 1987). Meanwhile, the AT is the pest density that consumes foliage or the proportion of infested plants that causes injury leading to yield loss in a crop with no reference to the economics of crop production or the cost of pest control (Harcourt et al. 1955, Shelton et al. 1983). The proportion of infested plants is the simplest and most commonly adopted AT for decision-making concerning the timing of insecticide application (Hamilton et al. 2009). The proportion of infested plants per sample AT estimated in this study (51%) was the same as those reported elsewhere (Shelton et al. 1983, Hamilton et al. 2009).

Chen and Su (1986) recommended a plant stage-specific larval density threshold of 1 and 2 larvae per plant, early and late in the season. However, in this study, we did not consider the plant growth stage but estimated an AT of 0.50 and 0.80 *P. xylostella* per plant/week for sampling during winter and spring, respectively. These thresholds are very similar and fall within the range of those reported by other studies (Mo and Baker 2004, Hamilton et al. 2008).

This study observed that *P. xylostella* exhibited distinct temporal patterns with an aggregated distribution in cabbage and satisfied all the components of an efficient sampling plan. The stop line for sampling charts requires higher sample sizes to estimate *P. xylostella* density. However, there is no practical difference in terms of the minimum sample size and pest action threshold. The sampling plans are therefore feasible for weekly monitoring because sample sizes are small even though the frequency may have to be increased during cup formation due to an upsurge in insect population during this period. The adoption and application of this sampling plan will reduce the frequency of chemical insecticide application and the associated detrimental effects.

## Data Availability

The data sets analyzed during the current study are available from the corresponding author on reasonable request.

## References

[CIT0001] Andaloro JT , Rose KB, Shelton AM, Hoy CW, Becker RF. Cabbage growth stages. N Y Food Life Sci Bull. 1983:101.

[CIT0002] Ayalew G. Comparison of yield loss on cabbage from diamondback moth, *Plutella xylostella* L. (Lepidoptera: Plutellidae) using two insecticides. Crop Prot. 2006:25(9):915–919. 10.1016/j.cropro.2005.12.001

[CIT0003] Ayalew G , Baumgärtner J, Ogol CKPO, Lohr B. Within-field spatial distribution and sampling plans for larvae and pupae of diamondback moth, *Plutella xylostella* L. (Lepidoptera: Plutellidae) in Ethiopian head cabbage field. Ethiop J Sci. 2006:29:149–156.

[CIT0004] Ayalew G , Sciarretta A, Baumgärtner J, Ogol C, Löhr B. Spatial distribution of diamondback moth, *Plutella xylostella* L. (Lepidoptera: Plutellidae), at the field and the regional level in Ethiopia. Int J Pest Manag. 2008:54(1):31–38. 10.1080/09670870701613743

[CIT0005] Binns MR , Nyrop JP, van der Werf W, Werf W. Sampling and monitoring in crop protection. Theoretical basis for developing practical decision guides. Wallingford (UK): CABI Publishing; 2000.

[CIT0006] Brenner RJ , Focks DA, Arbogast RT, Weaver DK, Human D. Practical use of spatial analysis in precision targeting for integrated pest management. Am Entomol. 1998:44:79–101.

[CIT0007] Brunce B. A class-analytic approach to Agricultural Joint Ventures in the communal areas of South Africa. STEPS Working Paper 103. Brighton (UK): STEPS Centre; 2018.

[CIT0008] Capinera JL. Handbook of vegetable pests. New York (NY): Academic Press; 2001.

[CIT0009] Cartwright B , Edelson JV, Chambers C. Composite action thresholds for the control of lepidopterous pests on fresh-market cabbage in the Lower Rio Grande Valley of Texas. J Econ Entomol. 1987:80:175–181.

[CIT0010] Charleston D. The tritrophic interactions of diamondback moth. Plant Prot News. 1998:51:8–9.

[CIT0011] Chen C , Su W. Spatial pattern and transformation of field counts of *Plutella xylostella* (L.) larvae on cauliflower. Plant Prot Bull. 1986:28:323–333.

[CIT0012] Chua TH , Lim BH. Distribution pattern of diamondback moth, *Plutella xylostella* (L.) (Lepidoptera: Plutellidae) on choy-sum plants. J Appl Entomol. 1979:88(1-5):170–175. 10.1111/j.1439-0418.1979.tb02492.x

[CIT0013] Dakshina S. Effectiveness of biological insecticides in controlling the diamondback moth (Lypidoptera: Plutellidae) using *Bacillus thuringiensis*, azadirachtin and new insecticides on cabbage. Fla State Hort Proc. 2013:126.

[CIT0014] Department of Agriculture, Forestry and Fisheries, South Africa. A profile of South African cabbage market value chain. Arcadia (South Africa): Department of Agriculture, Forestry and Fisheries; 2014.

[CIT0015] Francis RL , Smith JP, Shepard BM. Integrated pest management for cabbage and collard: a growers’ guide. Booklet No. EB 156. Clemson University PSA; 2005. p. 31.

[CIT0016] Furlong M , Ju K, Su P, Chol J, Il R, Zalucki MP. Integration of endemic natural enemies and *Bacillus thuringiensis* to manage insect pests of *Brassica* crops in North Korea. Agric Ecosyst Environ. 2008:125:223–238.

[CIT0017] Furlong MJ , Wright DJ, Dosdall LM. Diamondback moth ecology and management: problems, progress and prospects. Annu Rev Entomol. 2013:58:517–541. 10.1146/annurev-ento-120811-153605 23020617

[CIT0018] Furlong MJ , Zuhua Z, Shijian G, Yinquan L, Sheng LS, Zalucki MP. Quantitative evaluation of the biotic mortality factors affecting diamondback moth in southeast Queensland, Australia. In: Endersby N, Ridland PM, editors. The Management of Diamondback Moth and Other Crucifer Pests. Proceedings of the 4th International Workshop on Diamond Back Moth, 26–29 November 2001, Melbourne, Australia. Gosford (Australia): The Regional Institute; 2004. p. 185–194.

[CIT0069] Galvan TL , Burkness EC, Hutchison WD. Enumerative and binomial sequential sampling plans for the multicolored Asian lady beetle (Coleoptera: Coccinellidae) in wine grapes. J Econ Entomol. 2007:100:1000–1010. 17598567 10.1603/0022-0493(2007)100[1000:eabssp]2.0.co;2

[CIT0019] Grossrieder M , Kiefer B, Kang S, Kuhlmann U. Case study: knowledge transfer in cabbage IPM through farmer participatory training in DPR Korea. In: Hoddle MS, editor. Second International Symposium on Biological Control of Arthropods, 12–16 September 2005, Davos, Switzerland. Publication FHTET-2005-08. Washington (DC): USDA Forest Service; 2005. p. 318–332.

[CIT0020] Hamilton AJ , Endersby NM, Schellhorn NA, Ridland PM, Rogers PM, Jevremov D, Baker G. Evaluation of fixed sample-size plans for *Plutella xylostella* (Lepidoptera: Plutellidae) on broccoli crops in Australia. J Econ Entomol. 2006:99(6):2171–2176. 10.1603/0022-0493-99.6.2171 17195690

[CIT0021] Hamilton AJ , Hepworth G. Accounting for cluster sampling in constructing enumerative sequential sampling plans. J Econ Entomol. 2004:97(3):1132–1136. 10.1603/0022-0493(2004)097[1132:AFCSIC]2.0.CO;2 15279301

[CIT0022] Hamilton AJ , Waters EK, Kim HJ, Pak WS, Furlong MJ. Sampling a weighted pest complex: caterpillars in North Korean cabbage (*Brassica oleracea var. capitata*) crops. Entomol Exp Appl. 2008:130(3):282–289. 10.1111/j.1570-7458.2008.00815.x

[CIT0023] Hamilton AJ , Waters EK, Kim HJ, Pak WS, Furlong MJ. Validation of fixed sample size plans for monitoring lepidopteran pests of *Brassica oleracea* crops in North Korea. J Econ Entomol. 2009:102(3):1336–1343. 10.1603/029.102.0361 19610455

[CIT0024] Hammer O , Harper DAT, Ryan PD. PAST: paleontological statistics software package for education and data analysis. Palaeontol Electronica. 2001:4:9.

[CIT0025] Harcourt DG. Distribution of the immature stages of the diamondback moth, *Plutella maculipennis* (Curt.) (Lepidoptera: Plutellidae), on cabbage. Can Entomol. 1960:92(7):517–521. 10.4039/ent92517-7

[CIT0026] Harcourt DG , Backs RH, Cass LM. Abundance and relative importance of caterpillars attacking cabbage in eastern Ontario. Can Entomol. 1955:87(9):400–406. 10.4039/ent87400-9

[CIT0027] Hasanshahi G , Abbasipour H, Askarianzadeh A, Karimi J, Jahan F. Spatial summer–autumn distribution of the diamondback moth, *Plutella xylostella* (L.) and its parasitoids in cauliflower fields. Zool Ecol. 2017:27(1):57–63. 10.1080/21658005.2016.1261515

[CIT0028] Hodgson EW , Burkness EC, Hutchison WD, Ragsdale DW. Enumerative and binomial sequential sampling plans for soybean aphid (Homoptera: Aphididae) in soybean. J Econ Entomol. 2004:97(6):2127–2136. 10.1093/jee/97.6.2127 15666774

[CIT0029] Hutchison WD , Hogg DB, Poswal MAR, Berberet C, Cuperus GW. Implications of the stochastic nature of Kuno’s and Green’s fixed-precision stop lines: sampling plans for the pea aphid (Homoptera: Aphididae) in alfalfa as an example. J Econ Entomol. 1988:81:749–758.

[CIT0030] Iwao SA. New regression method for analyzing the aggregation pattern of animal populations. Res Popul Ecol. 1968:10:1–20.

[CIT0031] Kfir R. Effect of parasitoid elimination on populations of diamondback moth in cabbage. In: Endersby N, Ridland PM, editors. The Management of Diamondback Moth and Other Crucifer Pests, Proceedings of the 4th International Workshop on Diamond Back Moth, 26–29 November 2001, Melbourne, Australia. Gosford (Australia): The Regional Institute; 2004. p. 197–206.

[CIT0032] Kirby RD , Slosser JE. A composite economic threshold for three lepidopterous pests of cabbage. J Econ Entomol. 1984:77(3):725–733. 10.1093/jee/77.3.725

[CIT0033] Kuno E. A new method of sequential sampling to obtain the population estimates with a fixed level of precision. Res Popul Ecol. 1969:11(2):127–136. 10.1007/bf02936264

[CIT0034] Labou B , Brévault T, Sylla S, Diatte M, Bordat D, Diarra K. Spatial and temporal incidence of insect pests in farmers’ cabbage fields in Senegal. Int J Trop Insect Sci. 2017:37(04):225–233. 10.1017/s1742758417000200

[CIT0035] Lansink OA , Silva E. Non-parametric production analysis of pesticide use in the Netherlands. J Product Anal. 2004:21:49–65.

[CIT0036] Li J-Y , Chen Y-T, Shi M-Z, Li J-W, Xu R-B, Pozsgai G, You M-S. Spatio-temporal distribution patterns of *Plutella xylostella* (Lepidoptera: Plutellidae) in a fine-scale agricultural landscape based on geostatistical analysis. Sci Rep. 2021:11:13622. 34193887 10.1038/s41598-021-92562-9 PMC8245490

[CIT0037] Liebhold AM , Gurevitch J. Integrating the statistical analysis of spatial data in ecology. Ecography. 2002:25(5):553–557. 10.1034/j.1600-0587.2002.250505.x

[CIT0038] Lloyd M. Mean crowding. J Anim Ecol. 1967:36(1):1–30. 10.2307/3012

[CIT0039] Machekano H , Mvumi BM, Nyamukondiwa C. Diamondback moth, *Plutella xylostella* L. in Southern Africa: research trends, challenges and insights on sustainable management options. Sustainability. 2017:9(2):91–23. 10.3390/su9020091

[CIT0040] Marchioro C , Foerster L. Development and survival of the diamondback moth, *Plutella xylostella* (L.) (Lepidoptera: Yponomeutidae) as a function of temperature: effect on the number of generations in tropical and subtropical regions. Neotrop Entomol. 2011:40:533–541. 22068938

[CIT0041] Mo J , Baker G. Evaluation of sequential presence–absence sampling plans for the diamondback moth (Plutellidae: Lepidoptera) in cruciferous crops in Australia. J Econ Entomol. 2004:97(3):1118–1125. 10.1093/jee/97.3.1118 15279299

[CIT0042] Mosiane SM , Kfir R, Villet MH. Seasonal phenology of the diamondback moth, *Plutella xylostella* (L.), (Lepidoptera: Plutellidae), and its parasitoids on canola, *Brassica napus* (L.), in Gauteng province, South Africa. Afr Entomol. 2003:11:277–285.

[CIT0043] Ngowi BV , Tonnang HEZ, Mwangi EM, Johansson T, Ambale J, Ndegwa PN, Subramanian S. Temperature-dependent phenology of *Plutella xylostella* (Lepidoptera: Plutellidae): simulation and visualization of current and future distributions along the Eastern Afromontane. PLoS One. 2017:12(3):e0173590. 10.1371/journal.pone.0173590 28301564 PMC5354382

[CIT0044] Panthi BR , Renkema JM, Lahiri S, Liburd OE. Spatio-temporal distribution and fixed-precision sampling plan of *Scirtothrips dorsalis* (Thysanoptera: Thripidae) in Florida blueberry. Insects. 2021:12(3):256. 10.3390/insects12030256 33803537 PMC8002968

[CIT0045] Pezzini DT , DiFonzo CD, Finke DL, Hunt TE, Knodel JJ, Krupke CH, McCornack B, Michel AP, Moon RD, Philips CR, et al. Spatial patterns and sequential sampling plans for estimating densities of stink bugs (Hemiptera: Pentatomidae) in soybean in the North Central Region of the United States. J Econ Entomol. 2019:112(4):1732–1740. 10.1093/jee/toz100 31038178

[CIT0046] Picanço MC , Bacci L, Crespo ALB, Miranda MMM, Martins JC. Effect of integrated pest management practices on tomato *Lycopersicon esculentum*, production and preservation of natural enemies of pests. Agric For Entomol. 2007:9(4):327–335. 10.1111/j.1461-9563.2007.00346.x

[CIT0047] Quinn LP , de Vos BJ, Fernandes-Whaley M, Roos C, Bouwman H, Kylin H, Pieters R, van den Berg, J. Pesticide use in South Africa: one of the largest importers of pesticides in Africa. In: Stoytcheva M, editor. Pesticides in the modern world—pesticides use and management. Rijeka (Croatia): InTech; 2011. p. 49–96. https://www.dffe.gov.za/sites/default/files/docs/pesticide_usein_sa.pdf.

[CIT0068] Sarfraz M , Dosdall LM, Keddi BA. Fitness of parasitoid Diadegma insulare is affected by host's food plant. Basic Appl Ecol. 2009:10:563–572.

[CIT0048] Sereda B , Basson NCJ, Marais P. Bioassay of insecticide resistance in *Plutella xylostella* (L.) in South Africa. Afr Plant Prot. 1997:3:67–72.

[CIT0049] Shelton AM , Sears MK, Wyman JA, Quick TC. Comparison of action thresholds for lepidopterous larvae on fresh-market cabbage. J Econ Entomol. 1983:76(1):196–199. 10.1093/jee/76.1.196

[CIT0050] Shiojiri K , Takabayashi J, Yano S, Takafuji A. Oviposition preferences of herbivores are affected by tritrophic interaction webs. Ecol Lett. 2002:5(2):186–192. 10.1046/j.1461-0248.2002.00292.x

[CIT0051] Singh RV , Malik M, Kanojia AK, Singode A. A review paper on adoption behavior of vegetable growers towards pest management practices in Bulandshahr (U.P.), India. Int J Curr Microbiol Appl Sci. 2018:7(07):1364–1372.

[CIT0052] Southwood TRE. Ecological Methods: with particular reference to the study of insect populations. 2nd ed. London (UK): Chapman and Hall; 1978. p. 69.

[CIT0053] Southwood TRE , Henderson PA. Ecological methods. 4th ed. Hoboken (NJ): John Wiley and Sons; 2016.

[CIT0054] Sow G , Diarra K, Arvanitakis L, Bordat D. The relationship between the diamondback moth, climatic factors, cabbage crops and natural enemies in a tropical area. Folia Horticult. 2013:25(1):3–12. 10.2478/fhort-2013-0001

[CIT0055] Stabeno PJ , Schumacher JD, Bailey KM, Brodeur RD, Cokelet ED. Observed patches of walleye pollock eggs and larvae in Shelikof Strait, Alaska: their characteristics, formation and persistence. Fish Oceanogr. 1996:5:81–91.

[CIT0056] Statistic South Africa. Abstract agricultural statistics. Pretoria (South Africa): Department of Agriculture, Land Reform and Rural Development; 2021.

[CIT0057] Stemele MA. Impact of *Bacillus thuringiensis* Berliner var. *kurstaki* application on population densities of *Plutella xylostella* L. (Lepidoptera: Plutellidae), and its dominant parasitoid, *Cotesia vestalis* Haliday (Hymenoptera: Braconidae) and the implications on cabbage yield. Afr Entomol. 2016:24(2):398–406. 10.4001/003.024.0398

[CIT0058] Stemele MA. Comparative effects of selective insecticide, *Bacillus thuringiensis* var. *kurstaki* and broad-spectrum insecticide cypermethrin on diamondback moth and its parasitoid *cotesi vestalis* (hymenoptera; Braconidae). Crop Prot. 2017:101:35–42. 10.1016/j.cropro.2017.07.015

[CIT0059] Stern VM , Smith RF, van den Bosch R, Hage KS. The integration of chemical and biological control of the spotted alfalfa aphid. Part 1. The integrated control concept. Hilgardia. 1959:29:81–101.

[CIT0060] Talekar NS , Shelton AM. Biology, ecology, and management of the diamondback moth. Annu Rev Entomol. 1993:38(1):275–301. 10.1146/annurev.en.38.010193.001423

[CIT0061] Taylor LR. Assessing and interpreting the spatial distributions of insect populations. Annu Rev Entomol. 1984:29(1):321–357. 10.1146/annurev.en.29.010184.001541

[CIT0062] Uematsu H , Sakanoshita A. Micro-distribution of eggs of diamondback moth, *Plutella xylostella*, on intact and injured cabbage plantlets. Jpn J Appl Entomol Zool. 1993:37(1):1–3. 10.1303/jjaez.37.1

[CIT0063] Walsh B. Impact of insecticides on natural enemies in *Brassica* vegetables. Sydney (Australia): Horticulture Australia Ltd; 2005.

[CIT0064] Waters E , Hamilton AJ, Hepworth G, Kim H-J, Pak W-S, Furlong MJ. The influence of management practice on the spatial distribution of Lepidopteran pests in *Brassica* crops in the Democratic People’s Republic of Korea: implications for sequential sampling plans. Popul Ecol. 2009:51:525–531.

[CIT0065] Williamson S , Ball AS, Pretty J. Trends for pesticide use and safer pest management in four African countries. Crop Prot. 2008:27(10):1327–1334. 10.1016/j.cropro.2008.04.006

[CIT0066] Zalucki MP , Furlong MJ. Predicting outbreaks of a migratory pest: an analysis of DBM distribution and abundance. In: Shelton AM, Collins HL, Wu Q, Zhang Y, editors. Proceedings of the Fifth International Workshop on Diamondback Moth and Other Crucifer Pests, 24–27 October, Beijing, China. Beijing (China): China Agricultural Science and Technology Press; 2008. p. 453–454.

[CIT0067] Zalucki MP , Shabbir A, Silva R, Adamson D, Liu SS, Furlong MJ. Estimating the economic cost of one of the world’s major insect pests, *Plutella xylostella*: just how long is a piece of string? J Econ Entomol. 2012:105:1115–1129. 22928287 10.1603/EC12107

